# Social Determinants of Health and Other Predictors in Initiation of Treatment with CDK4/6 Inhibitors for HR+/HER2− Metastatic Breast Cancer

**DOI:** 10.3390/cancers16122168

**Published:** 2024-06-07

**Authors:** Ravi K. Goyal, Sean D. Candrilli, Susan Abughosh, Hua Chen, Holly M. Holmes, Michael L. Johnson

**Affiliations:** 1 Department of Pharmaceutical Health Outcomes and Policy, College of Pharmacy, University of Houston, Houston, TX 77004, USAmjohnso8@central.uh.edu (M.L.J.); 2 RTI Health Solutions, Research Triangle Park, NC 27709, USA; 3 Division of Geriatric and Palliative Medicine, McGovern Medical School, University of Texas, Houston, TX 78712, USA

**Keywords:** metastatic breast cancer, CDK4/6 inhibitors, palbociclib, social determinants of health, SDOH, Medicare, older patients

## Abstract

**Simple Summary:**

A new class of therapy named cyclin-dependent kinase 4/6 inhibitors (CDK4/6is) is the recommended preferred treatment for patients with metastatic breast cancer (MBC) who have the HR+/HER2− subtype; however, several barriers may still exist that prevent or delay the initiation of this treatment. In this observational study, we examined how the social determinants of health (SDOH) (e.g., income status, insurance coverage) and other patient characteristics are associated with the initiation of CDK4/6i for HR+/HER2− MBC in a Medicare population of patients aged 65 years or older. Our analysis showed that Medicare patients residing in areas with high vs. low median household income and those living in areas with a high vs. low proportion of Medicare-only coverage had higher rates of initiating treatment with CDK4/6i. Our study findings highlight the influence of SDOH on access to novel and effective cancer therapies and a need for strategies to improve equity in cancer care.

**Abstract:**

In hormone receptor-positive/human epidermal growth factor receptor 2-negative (HR+/HER2−) metastatic breast cancer (MBC), cyclin-dependent kinase 4/6 inhibitors (CDK4/6is) have replaced endocrine therapy alone as the standard of care; however, several barriers to treatment initiation still exist. We assessed social determinants of health (SDOH) and other factors associated with the initiation of CDK4/6i for HR+/HER2− MBC in the Medicare population. Using a retrospective cohort design, patients aged ≥65 years and diagnosed during 2015–2017 were selected from the SEER-Medicare database. Time from MBC diagnosis to first CDK4/6i initiation was the study outcome. The effect of SDOH measures and other predictors on the outcome was assessed using the multivariable Fine and Gray hazard modeling. Of 752 eligible women, 352 (46.8%) initiated CDK4/6i after MBC diagnosis (median time to initiation: 27.9 months). In adjusted analysis, SDOH factors significantly associated with CDK4/6i initiation included high versus low median household income (HHI) (hazard ratio [HR] = 1.70; 95% CI = 1.03–2.81) and the percentage of population with high versus low Medicare-only coverage (HR = 1.54; 95% CI = 1.04–2.27). In summary, older Medicare patients with HR+/HER2− MBC residing in areas with high median HHI and a high proportion of Medicare-only coverage had higher rates of initiating CDK4/6i, suggesting inequitable access to these novel, effective treatments and a need for policy intervention.

## 1. Introduction

The cyclin-dependent kinase 4 and 6 (CDK4/6) inhibitor therapies have transformed the treatment landscape in advanced and metastatic breast cancer (MBC), particularly for the molecular subtype that is defined by the hormone receptor-positive (HR+) and human epidermal growth factor receptor 2-negative (HER2−) status. Clinical trials of CDK4/6 inhibitors have demonstrated remarkable efficacy rates in terms of response rate and progression-free survival benefits [1,2,3]. The national clinical practice guidelines by the National Comprehensive Cancer Network (NCCN) have identified CDK4/6 inhibitors (palbociclib, ribociclib, and abemaciclib) in combination with endocrine therapy (ET) as the new standard of care and recommend them as the preferred frontline therapy on the basis of category 1 evidence for HR+/HER2− MBC [4] (NCCN makes no warranties of any kind whatsoever regarding their content, use, or application and disclaims any responsibility for their application or use in any way). 

Despite the recommendation, there are indications of potential suboptimal utilization of CDK4/6 inhibitors. After the initial growth in their uptake, recent reports indicate a plateaued trend, with no more than two-thirds of eligible women receiving treatment with this class of drugs [5,6]. A recent observational study based on a small, regional population of patients highlighted lower rates of CDK4/6 inhibitor therapy use by geographic region and income [5]. In another study of Medicare patients, CDK4/6 inhibitors were reported to be associated with substantial patient out-of-pocket costs [7]; the cost-sharing burden for novel anticancer medications is also known to be associated with delayed treatment initiation [8]. While an individual patient’s clinical characteristics, such as cancer stage, functional status, and presence of biomarkers or genetic mutations, play a central role in treatment selection, certain social determinants of health (SDOH) (e.g., race, marital status, geographic location) and enrollment characteristics (e.g., participation in the Low-Income Subsidy [LIS] program) may adversely affect the actual delivery of a guideline-concordant cancer treatment [9,10,11,12,13,14,15]. The characteristics of treatment with CDK4/6 inhibitors have been described previously in various populations [16,17,18]; however, limited data exist on factors that act as barriers, or facilitators, of treatment initiation with CDK4/6 inhibitors. Evidence is particularly scarce for the older population of patients in the US Medicare system who are generally aged 65 years or older. In this observational study, we used the Andersen’s Behavioral Model (ABM) of health services use—a widely adopted conceptual framework to analyze and predict the utilization of health services—to explore and describe the association between SDOH and the initiation of CDK4/6 inhibitor therapy for HR+/HER2− MBC at the Medicare population level. 

## 2. Materials and Methods

### 2.1. Study Design and Data Source

For this retrospective observational study, data were obtained from the Survey Epidemiology and End Results (SEER)-Medicare database (2015–2019). The SEER database captures detailed clinical information related to incident cancer cases from SEER registries. The SEER registries, implemented by the National Cancer Institute, are comprehensive reports tracking the incidence of cancer in selected regions that cover nearly 35% of the total US population. The SEER data are linked with the administrative medical and pharmacy claims data from the Medicare program and provide details on beneficiary demographics such as race, ethnicity, marital status, and geographic area of residence. The linkage is performed using the unique Social Security Numbers and individual names, and historically, nearly 96% of all newly diagnosed cancer patients aged 65 years or older are matched to Medicare data. These linked SEER-Medicare data are widely used to assess the profiles of patients diagnosed with cancer and their subsequent health care service utilization. The integrated Part D data capture detailed information on prescription drug utilization, including important health plan indicator variables that identify enrollment in the LIS program. Beneficiaries enrolled in the LIS program receive assistance with Medicare Part D costs like premiums, deductibles, copayments, and other prescription drug costs. The SEER-Medicare data were further combined with the SDOH database developed by the Agency for Healthcare Research and Quality to incorporate additional SDOH variables for this study [19]. Further details on the characteristics and applications of SEER-Medicare data, including generalizability of the analyses based on these data, have been described elsewhere [20].

### 2.2. Study Cohort

**Figure 1:**
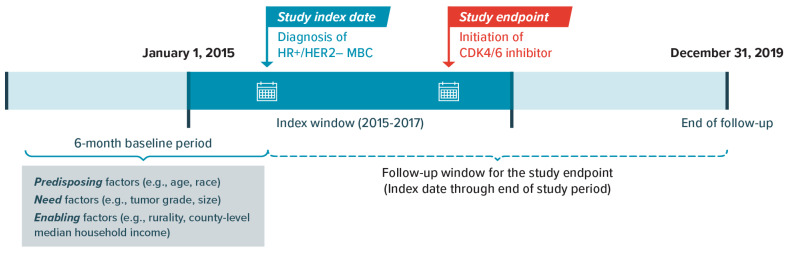
Study design schematic. CDK4/6 = cyclin-dependent kinase 4 and 6; HER2− = human epidermal growth factor receptor 2-negative; HR+ = hormone receptor-positive; MBC = metastatic breast cancer.

Women aged ≥65 years with confirmed diagnosis of HR+/HER2− MBC at initial presentation were selected. The population was restricted to patients with a diagnosis of MBC in or after 2015 to coincide with the introduction of the first CDK4/6 inhibitor. Eligible patients were also required to meet the following additional selection criteria: (1) breast cancer was their first or only primary cancer; (2) they initiated a systemic therapy within 12 months after the MBC diagnosis (systemic therapies [e.g., CDK4/6 inhibitors, ET, and chemotherapy] were selected using the treatment guidelines in effect during the study period); and (3) they had continuous enrollment in Medicare Part A (inpatient/hospital coverage) and Part B (outpatient/medical coverage), with no health maintenance organization (HMO) participation, for ≥6 months before the MBC diagnosis. Because CDK4/6 inhibitors are covered under the Medicare pharmacy benefit, patients were also required to have continuous enrollment in Part D (prescription drug coverage) during the follow-up period after MBC diagnosis. Patients were followed until death; loss of continuous coverage in Parts A, B, or D; enrollment in an HMO; or end of data (31 December 2019), whichever was earliest (Figure 1). This study was reviewed and deemed exempt by the University of Houston’s Institutional Review Board.

### 2.3. Study Variables

#### 2.3.1. Outcome

Initiation of treatment with any CDK4/6 inhibitor (alone or in combination) between 2015 and 2019 was the study outcome, defined as time-to-event measure, in months, from the date of MBC diagnosis to the date of first prescription for a CDK4/6 inhibitor. Patients who did not initiate a CDK4/6 inhibitor therapy during the study period were censored at the end of follow-up. Therapy lines and regimens containing CDK4/6 inhibitors were determined on the basis of methods used in previous work [21,22,23].

#### 2.3.2. Predictors

We did not specify a primary exposure and instead collectively assessed association of a prespecified set of SDOH measures and covariates with the outcome. A theoretical framework based on ABM of health services use was used for covariate classification [24]. According to the ABM, the *predisposing* factors included baseline patient characteristics (age at diagnosis, race, ethnicity, marital status, National Cancer Institute comorbidity index score for comorbidity burden, [25]). The *need* factors included tumor characteristics (tumor size at initial diagnosis, tumor grade, major sites of metastases [bone, brain, lung, and liver] identified from the SEER data). The *enabling* factors included health plan characteristics (LIS program enrollment), rural/urban status classified as large urban, small urban, and rural based on the rural–urban continuum codes, and select county-level SDOH measures, including median household income (HHI), percentage of non-English-speaking population, percentage of patients with some college education, and percentage of patients covered with Medicare-only insurance (no supplemental private insurance or other public insurance). All county-level measures were categorized on the basis of quartile distribution. The selection of reference category for covariates was informed by previous studies based on SEER-Medicare data and statistical considerations.

#### 2.3.3. Statistical Analysis

The baseline patient characteristics were first descriptively assessed. Time to first CDK4/6 inhibitor therapy was examined using Kaplan–Meier analysis and cumulative incidence function curve. To assess the association of potential predictors with the outcome, multivariable regression analysis was performed. Because many patients in our analysis were observed to die before initiating a CDK4/6 inhibitor therapy, an event of death was treated as a competing risk. In traditional Cox proportional hazard modeling, it is assumed that censoring is noninformative, i.e., censoring is unrelated or independent of the outcome and that censored patients could experience the event if they were observable beyond the point of censoring. However, in the case of death, this assumption of independent censoring does not hold, which generally leads to an overestimation of covariate effect. An appropriate alternative is subdistribution hazard modeling proposed by [26], which accounts for competing risks. The subdistribution hazard model is similar to the Cox proportional hazard model except that it models a hazard function that is derived from cumulative incidence function [27]. We estimated subdistribution hazard ratios (sHRs) and 95% confidence intervals (CIs) for all covariates, which were added to the model sequentially in sets of predisposing, need, and enabling factors. An examination of the model fit statistics using a likelihood ratio test showed that the addition of predictors from each set of predisposing, need, and enabling variables significantly improved the model fit. The sHR estimates were interpreted as relative rates [28]. All analyses were performed using SAS statistical software, version 9.4.

## 3. Results

### 3.1. Patient Characteristics

A total of 752 eligible women with de novo MBC diagnosis from 2015 to 2017 met the study selection criteria. The median age of the population was 75 years (range, 65–99); the majority were white (86.6%) and non-Hispanic (92.2%). Patients had a median follow-up time of 28 months (range, 1–60) since the MBC diagnosis. A detailed description of baseline demographics and clinical characteristics is provided in Table 1.

### 3.2. Treatment Characteristics

Overall, 352 patients (46.8%) initiated a systemic therapy containing a CDK4/6 inhibitor at any time after MBC diagnosis. Among these patients, palbociclib (92.1%, n = 324) was the most common CDK4/6 inhibitor, followed by ribociclib (4.8%, n = 17) and abemaciclib (3.1%, n = 11). By therapy line, 175 patients (23.3%) initiated CDK4/6 inhibitor therapy in the first line, of which 96.6% was in combination with ET. In the second line (n = 332), 160 patients (48.2%) received a CDK4/6 inhibitor therapy, of which 73.8% was in combination with ET and 23.8% was monotherapy. In the third line (n = 134), 51 patients (38.1%) received a CDK4/6 inhibitor therapy, of which 72.6% was in combination with ET and 21.6% was monotherapy.

**Figure 2:**
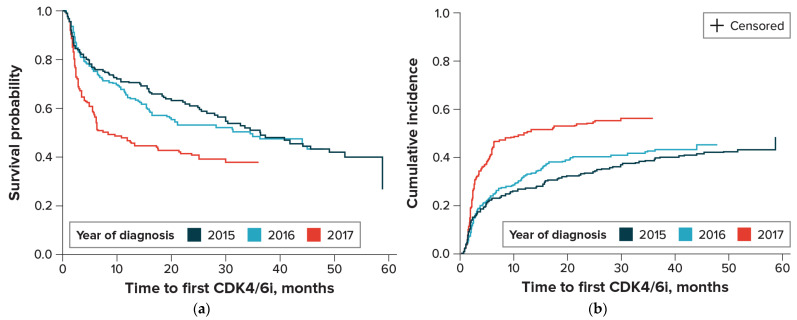
Time from MBC diagnosis to initiation of CDK4/6 inhibitor therapy, by year of diagnosis. CDK4/6 = cyclin-dependent kinase 4 and 6; MBC = metastatic breast cancer. (**a**) Kaplan–Meier Curve; (**b**) Cumulative Incidence Curve—adjusted for death as competing risk.

The estimated median time to first initiation of CDK4/6 inhibitor therapy, based on Kaplan–Meier analysis, was 27.9 months (95% CI, 20.0–34.6). Because the uptake of CDK4/6 inhibitors has increased over time, we also examined the time to initiation by year of diagnosis, and the estimated medians were as follows: 36.4 months (95% CI, 27.9–51.9) for 2015; 34.6 months (95% CI, 19.2–not estimable) for 2016; and 8.4 months (95% CI, 5.9–17.5) for 2017 (Figure 2a). In the bivariate model adjusted for competing risk, the cumulative incidence rate of CDK4/6 initiation at 24 months was 33.7% (95% CI, 27.8–39.8%) for 2015, 40.6% (95% CI, 34.4–46.6%) for 2016, and 54.3% (95% CI, 47.9–60.3%) for 2017 (Figure 2b).

### 3.3. Predictors of CDK4/6 Inhibitor Therapy Initiation

In the multivariable regression analysis (Table 1), the predisposing variables of age and year of diagnosis were found to be significant predictors of initiating treatment with CDK4/6 inhibitors. Patients in the age group ≥80 years had a significantly lower rate of treatment with CDK4/6 inhibitors compared with patients in the 65–69 years age group (sHR = 0.525; 95% CI, 0.375–0.734). Patients diagnosed with MBC in 2017 had a significantly higher rate of treatment compared with those diagnosed in 2015 (sHR = 1.669; 95% CI, 1.266–2.202). As compared with patients whose marital status was married at the time of diagnosis, those who were separated/divorced (sHR = 0.732; 95% CI, 0.505–1.061) had a lower rate of treatment with CDK4/6 inhibitors, although the effect was not statistically significant. No significant associations were seen for race (sHR = 0.958; 95% CI, 0.688–1.333) and ethnicity (sHR = 1.098; 95% CI, 0.675–1.785). Among the need variables, the presence of bone metastases at the time of diagnosis was associated with a significantly higher rate of treatment with CDK4/6 inhibitors compared with an absence of bone metastases (sHR = 1.343; 95% CI, 1.030–1.750). Of the enabling variables, median HHI and percentage of patients with Medicare only were associated with significantly higher rates. Women living in counties with higher median HHI had a significantly higher rate of treatment with CDK4/6 inhibitors versus those living in counties with lower median HHI (approximately 68% higher in income quartile 3 vs. income quartile 1 [sHR = 1.681; 95% CI, 1.122–2.519] and approximately 70% higher in income quartile 4 vs. income quartile 1 [sHR = 1.701; 95% CI, 1.030–2.811]). Moreover, the rate of CDK4/6 inhibitor therapy initiation was higher by nearly 54% in counties with a higher percentage of patients covered by Medicare only versus counties with a lower percentage of Medicare-only coverage (sHR = 1.535; 95% CI, 1.037–2.273).

## 4. Discussion

The findings from this study provide a detailed account of treatment initiation patterns of CDK4/6 inhibitors and offer insights into SDOH and other factors that predict rate of initiation among women with HR+/HER2− MBC in the Medicare population. Using an established conceptual framework of the ABM, this study highlights potential inequitable access to CDK4/6 inhibitors. According to the ABM, in an equitable environment, the utilization of health care services is driven predominantly by the need factors [24]; however, our analysis shows that the use of CDK4/6 inhibitors differed significantly by several predisposing factors, including age and year of diagnosis, and enabling factors comprising SDOH, namely, the median HHI and percentage of population covered by Medicare only. 

The finding that patients in the older age group (≥80 years) had a lower rate of CDK4/6 inhibitor therapy initiation than younger counterparts may be related to an increased risk of toxicity with CDK4/6 inhibitors that may be difficult to manage because of frailty, comorbidities, and comedications [29,30]. Year of diagnosis was found to be a key driver of treatment initiation with CDK4/6 inhibitors, with the most recent year (2017) having a significantly higher rate of use compared with 2015, when the CDK4/6 inhibitors first became available; this finding is likely a reflection of increased adoption of the therapy over time, as often seen when novel and effective therapies enter the market [31]. The significant increase in use in 2017 may also be driven partly by the fact that two new drugs in the class (ribociclib and abemaciclib) were approved in the same year, and associated media coverage and scientific discourse would have created increased awareness among providers. The finding of a higher rate of utilization in patients with bone metastases in our study is supported by a subgroup analysis based on the PALOMA trial, which concluded that the addition of palbociclib to letrozole led to clinically significant delays in the progression of bone metastases [32], although important differences exist between the current study and the PALOMA population based on baseline patient characteristics (e.g., age at diagnosis). The high cost of novel technologies (or drugs) is well established as a significant barrier to access. In the case of CDK4/6 inhibitors, a recent study showed that Medicare patients with standard prescription drug coverage experience substantial out-of-pocket costs for CDK4/6 inhibitors, which could extend as high as USD10,000 yearly [7]. It is established that high levels of patient cost-sharing responsibility for novel anticancer medications is associated with delayed treatment initiation [8]. In the present study, we found that women living in counties with high median HHI (>USD60,000) have significantly higher rates of treatment initiation with CDK4/6 inhibitors than those living in counties with low median HHI (≤USD45,000). This finding is consistent with the prior literature [9] and underscores the influence of SDOH on access to novel and effective cancer therapies. Additionally, the rate of treatment with CDK4/6 inhibitors was higher among women living in counties with a high percentage of the population insured by Medicare only (>4.9–15%) than among those living in counties with a low percentage of Medicare-only insurance (≤3.4%). This was an interesting finding with no direct explanation and is possibly related to the socioeconomic status of these geographies, i.e., a high percentage of persons with Medicare-only coverage would correspond with a low percentage of those with Medicaid-only or dual Medicare–Medicaid coverage, which is a commonly used proxy for social risk, with potentially reduced access to novel therapies [33,34]. 

Our findings align with previous reports of potentially suboptimal utilization of CDK4/6 inhibitors [5,6], with less than half of the patients being treated with these drugs in the early therapy lines despite evidence of clinical and survival benefits. Nevertheless, our analysis reports on the early period following the introduction of CDK4/6 inhibitors, and there is evidence of an increasing trend in utilization in the years following its introduction [35]. The observation period available for this study ended in December 2019. Since then, pivotal trials have reported evidence on survival benefits based on longer-term follow-ups, and those findings are expected to further influence the utilization patterns of CDK4/6 inhibitors in HR+/HER2− MBC.

Another interesting finding in our analysis was that a considerable proportion of patients (up to 24%) received CDK4/6 inhibitors as monotherapy, especially in the second- and third-line settings. This practice pattern suggests a lack of consistency with the treatment guidelines that recommend use of CDK4/6 inhibitors in combination with ET (i.e., with an aromatase inhibitor or an endocrine receptor down regulator), which is in line with the US Food and Drug Administration-approved indications. The only exception is abemaciclib, which is also approved for use as monotherapy in second or later lines. In a recent observational study based on electronic health records, use of abemaciclib as monotherapy was reported to be approximately 13% [17]. In another observational study, which predominantly consisted of palbociclib-treated patients, 15.4% of the patients were reported to have received CDK4/6 inhibitors as monotherapy [18].

There are certain limitations to consider in this study. As inherent in most observational studies based on claims databases developed for administrative purposes, any miscoding of procedure and drug codes may result in overestimation or underestimation of exposure and outcome. Even though the algorithm and rules to identify therapy lines and regimens were drawn from published methods [36], we presume some risk of misclassification. Further, as a limitation of the database, we did not have information on patients’ performance status and severity of the disease (i.e., presence of visceral crisis or rapid progression of disease), which may affect treatment choices. We also did not have any information on patient-level socioeconomic barriers to treatment initiation with CDK4/6 inhibitors, except for LIS enrollment. Nevertheless, we attempted to examine the effect of several SDOH predictors that were measured at the county level and highlighted key disparities that were observed according to the median HHI and percentage of Medicare-only population. Because the study sample size was dictated by the number of eligible patients available in the pre-existing database, we did not perform a formal sample size calculation; our study may be underpowered to sufficiently assess certain covariate effects. Further, our analysis was limited to patients with de novo metastatic disease (i.e., patients who progressed from an initial early-stage breast cancer to advanced/metastatic disease were not included), which potentially limits the generalizability our findings. Our findings may also not be representative of patients treated in settings outside of Medicare and those residing in countries outside of the US. Future research should investigate how the utilization of CDK4/6 inhibitors varies depending on the cancer stage at initial diagnosis as well as associations with clinical and socioeconomic risk factors that were unavailable for us to analyze in this study. Investigations in targeted therapies continue to transform the landscape for breast cancer care, and an ongoing assessment of barriers to diffusion and adoption of novel therapies, such as CDK4/6 inhibitors, is vital to understanding and improving quality of care for patients with MBC in one of the largest publicly insured populations in the United States.

## 5. Conclusions

In conclusion, the results of this observational study highlight that median HHI and percentage of the population with Medicare-only coverage were significant determinants of initiating treatment with a CDK4/6 inhibitor for HR+/HER2− MBC, underlining the broader issue of inequity in cancer care and influence of SDOH on access to novel and effective cancer therapies. Medicare policymakers should explore strategies to minimize disparities in access to these expensive but effective treatments. In the initial years since the availability of CDK4/6 inhibitors, our data indicate potentially suboptimal utilization, with only less than half of the patients receiving these treatments. As more patients receive these therapies over time, future research should investigate whether the noted disparities have narrowed and examine whether the predictors of utilization vary according to individual drugs in the CDK4/6 inhibitor class.

## Figures and Tables

**Table 1:** Multivariable Fine and Gray model assessing association of predictors on time to first CDK4/6 inhibitor therapy.

Predictors	Covariate Distribution		Multivariable Analysis ^a^		
	N	%	sHR	LCL	UCL
**Predisposing factors**					
Age at diagnosis, years					
65–69	174	23.14	Ref		
70–74	190	25.27	0.871	0.651	1.164
75–79	150	19.95	0.795	0.57	1.107
≥80	238	31.65	**0.525**	**0.375**	**0.734**
Race					
White	651	86.57	Ref		
Non-white	101	13.43	0.958	0.688	1.333
Ethnicity					
Non-Hispanic	708	94.15	Ref		
Hispanic	44	5.85	1.098	0.675	1.785
Marital status					
Married	285	37.9	Ref		
Separated/divorced	92	12.23	0.732	0.505	1.061
Single	96	12.77	1.029	0.746	1.420
Unknown	41	5.45	0.933	0.553	1.572
Widowed	238	31.65	0.844	0.625	1.139
Year of diagnosis					
2015	242	32.18	Ref		
2016	254	33.78	1.044	0.792	1.377
2017	256	34.04	**1.669**	**1.266**	**2.202**
Geographic region					
Midwest	82	10.9	Ref		
Northeast	192	25.53	0.818	0.418	1.600
South	207	27.53	1.194	0.68	2.099
West	271	36.04	0.846	0.513	1.395
**Need factors**					
Tumor grade					
1	94	12.5	Ref		
2	313	41.62	0.846	0.604	1.185
3	177	23.54	0.682	0.465	1.001
Unknown	168	22.34	0.818	0.545	1.229
Tumor size, cm					
0–2	99	13.16	Ref		
>2–4	217	28.86	0.895	0.596	1.344
>4–6	154	20.48	1.129	0.784	1.627
>6	143	19.02	1.04	0.703	1.537
Unknown	139	18.48	0.808	0.523	1.248
Involvement of metastatic site (yes/no; not mutually exclusive)					
Bone	538	71.54	**1.343**	**1.03**	**1.750**
Brain	35	4.65	0.746	0.413	1.347
Liver	113	15.03	0.984	0.723	1.339
Lung	218	28.99	1.122	0.871	1.445
NCI Comorbidity Index score					
0	456	60.64	Ref		
>0–1	238	31.65	0.923	0.719	1.185
>1	58	7.71	0.668	0.398	1.118
**Enabling factors**					
Rural/urban status					
Large urban	387	51.46	Ref		
Rural	236	31.38	1.207	0.747	1.948
Small urban	129	17.15	1.072	0.798	1.441
Any LIS coverage in last 6 months					
No	198	26.33	Ref		
Yes	554	73.67	0.817	0.61	1.093
Median household income					
≤USD45,000 (quartile 1)	172	22.87	Ref		
>USD45,000–USD60,000 (quartile 2)	173	23.01	1.371	0.942	1.996
>USD60,000–USD80,000 (quartile 3)	212	28.19	**1.681**	**1.122**	**2.519**
>USD80,000 (quartile 4)	195	25.93	**1.701**	**1.030**	**2.811**
Non-English-speaking population, %					
0 (quartile 1)	312	41.49	Ref		
>0–2 (quartile 2)	95	12.63	1.043	0.723	1.505
>2–8 (quartile 3)	165	21.94	1.307	0.945	1.806
>8 (quartile 4)	180	23.94	1.295	0.904	1.854
Persons with college education, %					
≤25 (quartile 1)	181	24.07	Ref		
>25–28 (quartile 2)	193	25.66	1.283	0.907	1.814
>28–32 (quartile 3)	184	24.47	0.873	0.587	1.297
>32 (quartile 4)	194	25.8	1.203	0.745	1.943
Persons covered with Medicare only, %					
≤3.4 (quartile 1)	186	24.73	Ref		
>3.4–4.2 (quartile 2)	189	25.13	0.969	0.674	1.394
>4.2–4.9 (quartile 3)	179	23.8	1.386	0.932	2.061
>4.9–15.0 (quartile 4)	198	26.33	**1.535**	**1.037**	**2.273**

CDK4/6 = cyclin-dependent kinase 4 and 6; ET = endocrine therapy; LCL = lower confidence limit; LIS = Low-Income Subsidy; NCI = National Cancer Institute; Ref = reference; SD = standard deviation; SEER = Survey Epidemiology and End Results; sHR = subdistribution hazard ratio; UCL = upper confidence limit. ^a^ Estimates in **bold** indicate statistically significant associations. Note: In compliance with SEER-Medicare Data Use Agreement, groups with frequencies less than 11 must be suppressed; therefore, data in some patient groups are collapsed together for reporting in this manuscript.

## Data Availability

The data that support the findings of this study are available from the National Cancer Institute. Restrictions apply to the accessibility of these data, which were purchased and used under license/data use agreement for this study.

## References

[1] Hortobagyi G.N., Stemmer S.M., Burris H.A., Yap Y.S., Sonke G.S., Hart L., Campone M., Petrakova K., Winer E.P., Janni W. (2022). Overall survival with ribociclib plus letrozole in advanced breast cancer. N. Engl. J. Med..

[2] Li Y., Li L., Du Q., Li Y., Yang H., Li Q. (2021). Efficacy and safety of CDK4/6 inhibitors combined with endocrine therapy in HR+/HER-2− ABC patients: A systematic review and meta-analysis. Cancer Investig..

[3] Piezzo M., Chiodini P., Riemma M., Cocco S., Caputo R., Cianniello D., Di Gioia G., Di Lauro V., Di Rella F., Fusco G. (2020). Progression free survival and overall survival of CDK 4/6 inhibitors plus endocrine therapy in metastatic breast cancer: A systematic review and meta-analysis. Int. J. Mol. Sci..

[4] National Comprehensive Cancer Network (NCCN) (2020). Clinical Practice Guidelines in Oncology: Breast Cancer. Version 5. https://www2.tri-kobe.org/nccn/guideline/breast/english/breast.pdf.

[5] Lee K.T., Chiao E., Lim D., Mouslim M., Wang C., Mangini N., Stearns V., Smith K.L. Predictors of non-receipt of first-line CDK 4/6 inhibitors (CDK4/6i) among patients with metastatic breast cancer (MBC). Proceedings of the 2021 American Society of Clinical Oncology (ASCO) Annual Meeting.

[6] (2021). New studies reveal potential mechanisms of resistance to CDK4/6 inhibitors in HR+/HER2- breast cancer. Oncologist.

[7] Golbach A.P., Smith M.D., Taraba J.L., Giridhar K.V. (2021). Abstract PS7-49: Assessment of patient out-of-pocket cost for palbociclib therapy in advanced breast cancer. Cancer Res..

[8] Doshi J.A., Li P., Huo H., Pettit A.R., Armstrong K.A. (2018). Association of patient out-of-pocket costs with prescription abandonment and delay in fills of novel oral anticancer agents. J. Clin. Oncol..

[9] Vyas A., Gabriel M., Kurian S. (2021). Disparities in guideline-concordant initial systemic treatment in women with HER2-negative metastatic breast cancer: A SEER-Medicare analysis. breast cancer: Targets and therapy. Breast Cancer.

[10] Chou Y.T., Farley J.F., Stinchcombe T.E., Proctor A.E., Lafata J.E., Dusetzina S.B. (2020). The association between Medicare low-income subsidy and anticancer treatment uptake in advanced lung cancer. J. Natl. Cancer Inst..

[11] Cobran E.K., Chen R.C., Overman R., Meyer A.M., Kuo T.M., O’Brien J., Sturmer T., Sheets N.C., Goldin G.H., Penn D.C. (2016). Racial differences in diffusion of intensity-modulated radiation therapy for localized prostate cancer. Am. J. Mens Health.

[12] Pisu M., Oliver J.S., Kim Y.I., Elder K., Martin M., Richardson L.C. (2010). Treatment for older prostate cancer patients: Disparities in a southern state. Medical Care.

[13] Lund M.J., Brawley O.P., Ward K.C., Young J.L., Gabram S.S., Eley J.W. (2008). Parity and disparity in first course treatment of invasive breast cancer. Breast Cancer Res. Treat..

[14] Griggs J.J., Culakova E., Sorbero M.E., Poniewierski M.S., Wolff D.A., Crawford J., Dale D.C., Lyman G.H. (2007). Social and racial differences in selection of breast cancer adjuvant chemotherapy regimens. J. Clin. Oncol..

[15] Haggstrom D.A., Quale C., Smith-Bindman R. (2005). Differences in the quality of breast cancer care among vulnerable populations. Cancer.

[16] Law J.W., Mitra D., Kaplan H.G., Alfred T., Brufsky A.M., Emir B., McCracken H., Liu X., Broome R.G., Zhang C. (2022). Real-world treatment patterns and clinical effectiveness of palbociclib plus an aromatase inhibitor as first-line therapy in advanced/metastatic breast cancer: Analysis from the US Syapse Learning Health Network. Curr. Oncol..

[17] Cuyun Carter G., Sheffield K.M., Gossai A., Huang Y.J., Zhu Y.E., Bowman L., Nash Smyth E., Mathur R., Cohen A.B., Rasmussen E. (2021). Real-world treatment patterns and outcomes of abemaciclib for the treatment of HR+, HER2− metastatic breast cancer. Curr. Med. Res. Opin..

[18] Husinka L., Koerner P.H., Miller R.T., Trombatt W. (2021). Review of cyclin-dependent kinase 4/6 inhibitors in the treatment of advanced or metastatic breast cancer. J. Drug Assess..

[19] Agency for Healthcare Research and Quality (2022). Social Determinants of Health Database.

[20] Enewold L., Parsons H., Zhao L., Bott D., Rivera D.R., Barrett M.J., Virnig B.A., Warren J.L. (2020). Updated overview of the SEER-Medicare data: Enhanced content and applications. J. Natl. Cancer Inst. Monogr..

[21] Goyal R.K., Cuyun Carter G., Nagar S.P., Nash Smyth E., Price G.L., Parikh R.C., Huang Y.J., Li L., Davis K.L., Kaye J.A. (2021). Treatment patterns, adverse events, and direct and indirect economic burden in a privately insured population of patients with HR+/HER2–metastatic breast cancer in the United States. Expert Rev. Pharmacoecon. Outcomes Res..

[22] Goyal R.K., Carter G.C., Nagar S.P., Smyth E.N., Price G.L., Huang Y.J., Li L., Davis K.L., Kaye J.A. (2019). Treatment patterns, survival and economic outcomes in Medicare-enrolled, older patients with HR+/HER2− metastatic breast cancer. Curr. Med. Res. Opin..

[23] Davis K.L., Goyal R.K., Able S.L., Brown J., Li L., Kaye J.A. (2015). Real-world treatment patterns and costs in a US Medicare population with metastatic squamous non-small cell lung cancer. Lung Cancer.

[24] Andersen R.M. (1995). Revisiting the behavioral model and access to medical care: Does it matter?. J. Health Soc. Behav..

[25] Klabunde C.N., Legler J.M., Warren J.L., Baldwin L.M., Schrag D. (2007). A refined comorbidity measurement algorithm for claims-based studies of breast, prostate, colorectal, and lung cancer patients. Ann. Epidemiol..

[26] Fine J.P., Gray R.J. (1999). A proportional hazards model for the subdistribution of a competing risk. J. Am. Stat. Assoc..

[27] Austin P.C., Lee D.S., Fine J.P. (2016). Introduction to the analysis of survival data in the presence of competing risks. Circulation.

[28] Austin P.C., Fine J.P. (2017). Practical recommendations for reporting fine-gray model analyses for competing risk data. Stat. Med..

[29] Ismail R.K., van Breeschoten J., Wouters M.W., van Dartel M., van der Flier S., Reyners A.K., de Graeff P., Pasmooij A.M., de Boer A., Broekman K.E. (2021). Palbociclib dose reductions and the effect on clinical outcomes in patients with advanced breast cancer. Breast.

[30] Howie L.J., Singh H., Bloomquist E., Wedam S., Amiri-Kordestani L., Tang S., Sridhara R., Sanchez J., Prowell T.M., Kluetz P.G. (2019). Outcomes of older women with hormone receptor–positive, human epidermal growth factor receptor–negative metastatic breast cancer treated with a CDK4/6 inhibitor and an aromatase inhibitor: An FDA pooled analysis. J. Clin. Oncol..

[31] O’Connor J.M., Fessele K.L., Steiner J., Seidl-Rathkopf K., Carson K.R., Nussbaum N.C., Yin E.S., Adelson K.B., Presley C.J., Chiang A.C. (2018). Speed of adoption of immune checkpoint inhibitors of programmed cell death 1 protein and comparison of patient ages in clinical practice vs pivotal clinical trials. JAMA Oncol..

[32] Finn R.S., Crown J., Lang I., Kulyk S.O., Schmidt M., Patel R., Thummala A., Bondarenko I., Randolph S., Kim S. (2015). The effect of palbociclib (P) in combination with letrozole (L) on bone metastases in women with ER+/HER2-metastatic breast cancer (MBC): Subanalysis from a randomized phase II study. J. Clin. Oncol..

[33] Office of the Assistant Secretary for Planning and Evaluation (ASPE) (2016). Report to Congress: Social Risk Factors and Performance Under Medicare’s Value-Based Purchasing Programs. https://aspe.hhs.gov/sites/default/files/private/pdf/253976/RTCAppendices.pdf.

[34] Rosenbaum S. (2014). Medicaid payments and access to care. N. Engl. J. Med..

[35] Goyal R.K., Holmes H.M., Chen H., Abughosh S., Candrilli S.D., Johnson M.L. (2023). Impact of CDK4/6 inhibitors on chemotherapy utilization in earlier therapy lines for HR+/HER2–metastatic breast cancer in the United States. Breast Cancer Res. Treat..

[36] Goyal R.K., Chen H., Abughosh S.M., Holmes H.M., Candrilli S.D., Johnson M.L. (2023). Overall survival associated with CDK4/6 inhibitors in patients with HR+/HER2–metastatic breast cancer in the United States: A SEER-Medicare population-based study. Cancer.

